# Vascular Endothelial Growth Factor Signaling in Models of Oxygen-Induced Retinopathy: Insights Into Mechanisms of Pathology in Retinopathy of Prematurity

**DOI:** 10.3389/fped.2021.796143

**Published:** 2021-12-09

**Authors:** Aniket Ramshekar, M. Elizabeth Hartnett

**Affiliations:** Department of Ophthalmology and Visual Sciences, John A. Moran Eye Center, University of Utah, Salt Lake City, UT, United States

**Keywords:** ROP, OIR, VEGF, VEGFRs, VEGF receptors, neuropilins

## Abstract

Retinopathy of prematurity (ROP) is a leading cause of blindness in children worldwide. Blindness can occur from retinal detachment caused by pathologic retinal angiogenesis into the vitreous, termed intravitreal neovascularization (IVNV). Although agents that interfere with the bioactivity of vascular endothelial growth factor (VEGF) are now used to treat IVNV, concerns exist regarding the identification of optimal doses of anti-VEGF for individual infants and the effect of broad VEGF inhibition on physiologic angiogenesis in external organs or in the retina of a preterm infant. Therefore, it is important to understand VEGF signaling in both physiologic and pathologic angiogenesis in the retina. In this manuscript, we review the role of receptors that interact with VEGF in oxygen-induced retinopathy (OIR) models that represent features of ROP pathology. Specifically, we discuss our work regarding the regulation of VEGFR2 signaling in retinal endothelial cells to not only reduce severe ROP but also facilitate physiologic retinal vascular and neuronal development.

## Introduction

Retinopathy of prematurity (ROP) remains a leading cause of blindness in children worldwide despite advances in neonatal care (1). The pathophysiology of ROP is described by a two-phase hypothesis that has been refined with the ability to save extremely premature infants (2). In Phase I ROP, intraretinal vascularization is compromised, and ongoing physiologic vascular development is delayed leading to areas of hypoxic retina. In Phase II ROP, also classified as Stage 3 ROP (3), aberrant retinal angiogenesis grows into the vitreous and is termed intravitreal neovascularization (IVNV). IVNV leads to blindness from retinal detachment that is not, or cannot be, treated (4, 5). Currently, Phase II ROP is treated with methods to ablate the peripheral avascular retina, often with laser (6), or with intravitreal agents that interfere with the bioactivity of vascular endothelial growth factor (VEGF) (7–11). However, broad inhibition of VEGF in preterm infants might interfere with physiologic angiogenesis in external organs or in the developing retina where it can lead to persistent avascular retina and recurrent IVNV (9). Understanding VEGF-mediated molecular mechanisms involved in IVNV and physiologic vascular development of the peripheral retina is important to identify safe and effective treatment targets.

**Figure 1:**
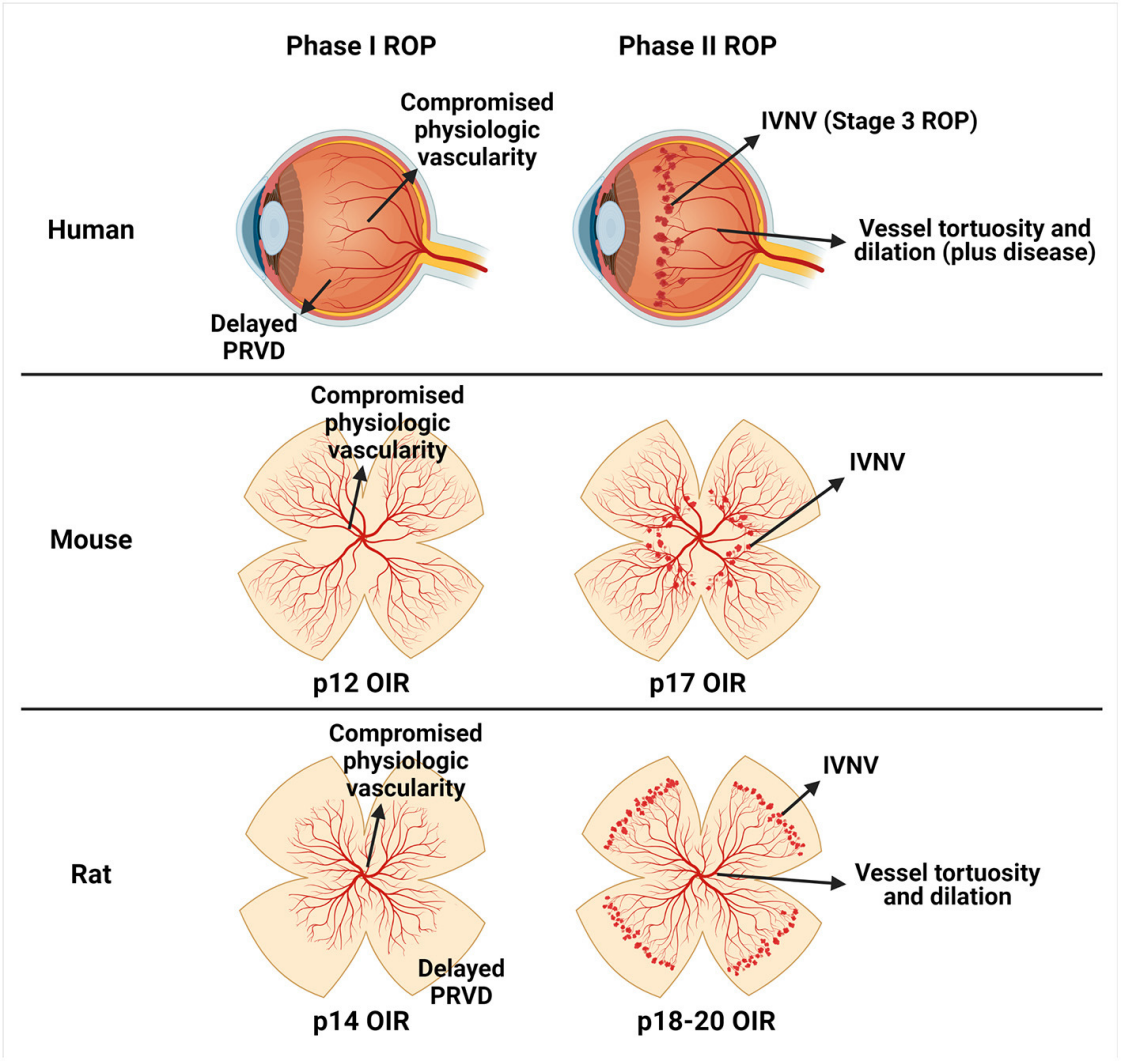
Schematic representation of similarities between human retinopathy of prematurity (ROP) and oxygen-induced retinopathy (OIR) models. Human ROP is described by a two-phase hypothesis (row 1). In Phase I, events surrounding preterm birth (i.e., lack of maternally derived factors, relative hyperoxia, repeated oxygen fluctuations, poor infant growth, etc.) cause a delay in physiologic retinal vascular development (PRVD) and compromise to already developed vessels (compromised physiologic vascularity). In Phase II, the hypoxic avascular retina releases pro-angiogenic factors that promote aberrant retinal angiogenesis into the vitreous termed intravitreal neovascularization (IVNV). The murine OIR model (row 2) recapitulates Phase I compromised physiologic vascularity and has been termed vaso-obliteration at p12, and Phase II IVNV at p17. The rat OIR model (row 3) recapitulates Phase I delay in PRVD to the peripheral retina and compromised physiologic vascularity at p14, and Phase II IVNV and vessel tortuosity and dilation at p18-20. Created with Biorender.com.

To understand the role of VEGF in the pathophysiology of ROP, studies were conducted using animal models of oxygen-induced retinopathy (OIR) that recapitulate features of ROP pathology in preterm infants. The most common models were in mouse, rat, and beagle (5). The models differ based on the extent of inner vascular plexus coverage to the ora serrata at the time animals are placed into the model, oxygen levels, duration of exposure to oxygen, the age when animals are placed into the model, and the features of ROP represented by in the model. In the murine OIR model, mice are born and raised in room air until postnatal day (p)7 when intraretinal vascularization of the inner plexus extends to the ora serrata. At p7, mice are placed into 75% oxygen for 5 days, which causes hyperoxia-induced compromise of the developed inner plexus in the central retina surrounding the optic nerve (vaso-obliteration). Mice are returned to room air and develop preretinal neovascular tufts (IVNV) at the junction of the vascular and avascular retina at p17 (Phase II) (12). In the rat model, newborn rat pups with almost no retinal vascular development are exposed to oxygen extremes that fluctuate between 50% and 10% every 24 h for 14 days. At p14, rats have compromised physiologic vascularity and delayed physiologic retinal vascular development (Phase 1). Pups are placed into room air and develop IVNV at p18-20 (Phase II) (13). Although the mouse model is often used for ease of genetic manipulation, the rat OIR model best represents human ROP based on oxygen stresses similar to those in preterm infants (fluctuations in oxygen and changes in extremes of arterial oxygen), similar appearing Phases in ROP (Phase I compromise in physiologic vascularization and delay in physiologic vascular development of the peripheral retina at p14, and Phase II IVNV, vascular tortuosity, and vascular dilation at p18-20), and extrauterine growth restriction (Figure 1). In the beagle OIR model, newborn pups at p1 are placed into 100% oxygen for 4 days and, at p5, are returned to room air. The beagle OIR model develops delayed physiologic retinal vascular development and compromised physiologic vascularity (Phase I) and IVNV (Phase II) that have been measured at p15 and observed until p45 (14–16). This OIR model is useful to assess pharmacologic treatments due to increased eye size in beagles compared with rodents.

There are five members of the VEGF family: VEGFA, placental growth factors (PlGFs), VEGFB, VEGFC, and VEGFD (17). Studying the role of VEGF in the murine OIR model is difficult since a single allele knockout of VEGF or VEGF receptors (VEGFRs) is lethal in mice (18–20). Although transgenic mice lacking VEGFB (*Vegfb*^−/−^) are viable, no difference was observed in IVNV compared with littermate wild-type mice (21). In rat pups raised in OIR compared with room air, retinal VEGFA protein was significantly increased and, mainly, VEGFA splice variant 164 (VEGFA_164_) mRNA at p14 and p18 (22–24). These findings implicated VEGFA in both physiologic retinal vascular development and IVNV. Therefore, broad inhibition of VEGFA was predicted to reduce both. Surprisingly, intravitreal neutralizing antibodies to rat VEGFA compared with IgG significantly reduced IVNV in a dose-dependent manner without interfering with physiologic retinal vascular development at p18 in rat pups. However, IVNV and avascular retina area within the vascularized retina were significantly increased at p25 in rat pups that received an effective dose of anti-VEGFA (25). A VEGF-Trap, which binds VEGFA and PlGFs, was compared with a human Fc control after intravitreal injection at p8 in beagle pups. At p21, both IVNV and physiologic retinal vascular development were reduced at high doses of the VEGF-Trap compared with control. However, the lowest dose (5 μg) of the VEGF-Trap reduced IVNV but not physiologic retinal vascular development (26). Taken together, these studies provide experimental evidence that anti-VEGF agents can interfere with physiologic retinal vascular development, compromise already developed retinal vasculature, and lead to recurrent IVNV at certain doses. Therefore, studies were warranted to refine the dose of anti-VEGF agents that would inhibit IVNV and permit sufficient VEGF expression at a concentration that allows physiologic vascular development of the peripheral retina. In support of this notion, Müller cells or astrocytes in the retina were demonstrated to overproduce VEGFA implicated in the development of IVNV in the murine OIR model (27–29). In rat pups raised in the OIR model, novel approaches to knock down VEGFA or VEGFA_164_ in Müller cells by subretinal introduction of lentiviral vectors that contained a CD44 promoter upstream of an miR-30-based shRNA cassette significantly reduced IVNV at p18 (30, 31) without recurrence at p32 (32). However, lentiviral-mediated knockdown of Müller cell-derived VEGFA thinned the retinal outer nuclear layer compared with knockdown of Müller cell-derived VEGFA_164_ by lentiviral vectors (30). Although the data supported the hypothesis that an optimal anti-VEGF dose will not interfere with physiologic vascular development of the peripheral retina, identifying this dose in infants might be challenging due to variation of pathology among individual infants or eyes (33). Nonetheless, the data support the involvement of VEGFA in the pathophysiology of ROP and physiologic development of retinal vasculature, neurons, and glia. In this article, we discuss VEGFA signaling through different receptors in models of ROP to identify mechanisms involved in the Phases of ROP pathology and provide insights into novel therapeutic approaches for ROP that overcome limitations in identifying optimal doses of antiangiogenic agents for individual infants.

## The Role of Vascular Endothelial Growth Factor Receptors in Models of Retinopathy of Prematurity

VEGF members bind to VEGF receptors (VEGFRs), which induce receptor homodimerization or heterodimerization and activation through autophosphorylation of the tyrosine residues in the receptor intracellular domains (34). There are three subtypes of VEGFRs, but VEGFA binds VEGFR1 or VEGFR2 to elicit biologic functions (35). Immunohistochemical staining of retinal sections from mice in the OIR model demonstrated colocalization of von Willebrand factor-labeled IVNV and VEGFR2, but not VEGFR1, at p19 (36). Retinal lysates from p18 rat pups raised in the OIR model had increased VEGFR2 mRNA, but not VEGFR1 mRNA, compared with p18 pups raised in room air (24). Immunostaining of retinal sections from rats raised in OIR demonstrated immunolabeling of VEGFR1 and VEGFR2 in areas of IVNV at p20 (37). Colocalization of VEGFR2 and von Willebrand factor-stained IVNV was also observed in retinal sections from p15 dogs raised in OIR (16). Specifically, immunostaining of phosphorylated VEGFR2 was reduced in retinal sections from p13 rats that were raised in rat OIR and treated with intravitreal antibodies against VEGFA compared with IgG (23). These findings primarily implicated VEGFR2 in the pathophysiology of ROP; however, this review will summarize studies regarding the role of VEGFR1 and VEGFR2 in models of ROP.

### The Role of Vascular Endothelial Growth Factor Receptor 1 in Models of Retinopathy of Prematurity

Intraperitoneal administration of antibodies against VEGFR1 compared with IgG in mice reduced IVNV in mice placed in OIR (38, 39). However, intravitreal PlGF1, a VEGFR1-specific ligand, resulted in no difference in IVNV compared with buffered salt solution control even though previous investigators reported reduced IVNV after intravitreal neutralizing antibody to VEGFR1 (40). The disparity in studies might be because PlGF1 does not bind VEGFR2 monomers (41), and VEGFR2-related signaling is important in IVNV (see *The role of vascular endothelial growth factor receptor 2 in models of retinopathy of prematurity* section). In support of this notion, Zeng et al. observed disordered divisions of mouse embryonic stem cell-derived vessels from VEGFR1 knockout mice (*flt*1^−/−^) (42). VEGFR1 acts as a decoy receptor, and when knocked out, it does not bind to VEGF, which permits more VEGF to trigger signaling through VEGFR2 (43). In line with this thinking, rescue of VEGFR1 expression in *flt*1^−/−^ embryonic stem cell-derived vessels, with a transgene that expressed soluble VEGFR1 under the guidance of a PECAM promoter, reduced randomized divisions of endothelial cells and increased ordered divisions (42). Similarly, in the rat OIR model, pups treated with intravitreal anti-VEGFA antibodies had significantly more vascular cell divisions that favored vascular extension rather than widening (44). The studies provided strong evidence that regulation of VEGFR2 is important in orienting dividing endothelial cells and supports the hypothesis that ordered divisions extend peripheral vascular development that occurs in developing retina. The role of VEGFR1 activation in physiologic vascular development of the peripheral retina using a representative model of ROP remains unknown.

### The Role of Vascular Endothelial Growth Factor Receptor 2 in Models of Retinopathy of Prematurity

As indicated in the above studies (40, 42), evidence suggested a role for VEGFR2 in ROP. Further support was found in mice with significantly reduced IVNV after gavage with an antagonist to VEGFRs and platelet-derived growth factor receptors (PDGFRs, PTK787) compared with selective PDGFR antagonists (CGP57148 or CGP53716) or vehicle control (45). Similarly, mice treated with a subcutaneous tyrosine kinase inhibitor (SU5416) had significantly reduced IVNV. However, room air-raised mice treated with SU5416 compared with vehicle control had significantly reduced intraretinal vascular extension of the inner plexus to the ora serrata and reduced total retinal thickness of the peripheral retina (46). OIR-raised dogs implanted with a pellet that released antibodies against VEGFR2 into the vitreous had significantly reduced IVNV and delayed physiologic vascular development of the peripheral retina compared with pups implanted with a pellet that released IgG into the vitreous (16). Taken together, the data suggest that inhibition of VEGFR2 affects both physiologic and pathologic retinal angiogenesis and retinal structure. Therefore, this approach might not be a safe therapy for ROP. In an effort to regulate VEGFR2 signaling specifically in retinal endothelial cells, lentiviral vectors that expressed shRNA against VEGFR2 or luciferase control under the guidance of an endothelial-specific promoter, *Cdh5*, were tested in the rat OIR model. Knock down of VEGFR2 in endothelial cells by shRNA significantly reduced IVNV and allowed more physiologic vascular development of the peripheral retina compared with littermate controls at p20. Furthermore, total retinal thickness near the optic nerve head was not thinned after lentiviral delivered *Cdh5*-targeted shRNA against VEGFR2 compared with littermate controls. There was also no difference in a- or b-wave amplitudes assessed by full-field electroretinography in adult rats compared with littermate controls (47). These findings contrasted with earlier studies in which Müller cell-derived VEGFA knockdown by lentiviral vectors in the rat OIR led to retinal thinning, (32) and intravitreal VEGF-Trap delayed physiologic retinal vascular development in the dog OIR model (26) compared with respective controls. Taken together, the data support the thinking that VEGFA signaling is important for neural retinal structure and function, and normal retinal vascularization. Furthermore, regulation of VEGFR2 signaling in retinal endothelial cells accomplishes safe inhibition of IVNV while promoting physiologic retinal vascular development and retinal structure and function. The data also suggest that a certain dose or agent that regulates VEGF-mediated signaling triggered through VEGFR2 in retinal endothelial cells might be a possible therapeutic approach to inhibit IVNV, facilitate physiologic retinal vascular development, and reduce the likelihood of recurrent IVNV in ROP.

## The Role of Neuropilins in Models of Retinopathy of Prematurity

Originally identified in *Xenopus* tadpole nervous tissues (48) as receptors for semaphorins (49, 50), neuropilins are cell surface glycoproteins that bind to VEGF family members (51) and form complexes with VEGFRs as co-receptors (52). There are two isoforms of the protein, neuropilin 1 and neuropilin 2, and both have been demonstrated to interact with VEGFRs to trigger signaling induced by VEGFA. Also, VEGFA_164_ has been demonstrated to bind to neuropilin 1 and neuropilin 2 (53). Neuropilin 1 mRNA was increased in retinal lysates from mice placed in OIR compared with room air at p17 (54). Also at p17, retinal sections from mice placed in OIR demonstrated colocalization of neuropilin 1 mRNA with IVNV (55). Specifically, neuropilin 1 (54, 56) or neuropilin 2 (57) protein colocalized with IVNV. Furthermore, Budd et al. found significantly increased neuropilin 1 and neuropilin 2 mRNA in rats raised in OIR compared with room air at p14 and p18 (58).

### The Role of Neuropilin 1 in Models of Retinopathy of Prematurity

Neuropilin 1 knockout mice (*Nrp*1^−/−^) are embryonically lethal (59–61). Neutralizing neuropilin 1 with intravitreal antibody significantly reduced IVNV compared with IgG in mouse OIR (55). Compared with littermate control mice that lacked Cre alleles, tamoxifen-inducible knock out of endothelial neuropilin 1 in a Cre-loxP mouse model reduced IVNV in mice in OIR and delayed intraretinal vascular development of the inner plexus in mice raised in room air (62). However, knock out of neuropilin 1 in myeloid lineage cells using *LysM*-Cre did not affect intraretinal vascular development of the inner plexus in room air compared with mice that lacked the floxed *Nrp1* alleles but still expressed *LysM*-Cre (63). These findings implicate endothelial neuropilin 1 not only in the development of IVNV but also in physiologic retinal vascular development.

To understand mechanistically how neuropilin 1 regulates angiogenesis, transgenic mice that expressed a mutant neuropilin 1 that lacked the cytoplasmic domain of the receptor were generated (64). The cytoplasmic domain of neuropilin 1 has been reported to interact with VEGFR2 to enhance VEGFR2-mediated signaling (65–67). Therefore, expression of a mutant neuropilin 1 receptor that lacked the ability to interact with VEGFR2 to trigger signaling might affect intraretinal vascular development in mice. However, the study reported no difference in intraretinal vascular development of the inner plexus between room air raised mice that expressed a mutant neuropilin 1 and littermate control mice that expressed wild-type neuropilin 1 (64). To determine if VEGFA-binding neuropilin 1 was required for angiogenesis, transgenic mice that expressed a mutant neuropilin 1 with a point mutation in the VEGF-binding b1 domain (*Nrp*1^Y297A/Y297A^) were generated along with littermate wild-type controls. *Nrp*1^Y297A/Y297A^ mice raised in room air had significantly reduced intraretinal vascular extension of the inner plexus at p7 and reduced IVNV in OIR at p17 compared with age-controlled littermate wild-type mice (68). Taken together, these observations suggest that VEGF-binding endothelial neuropilin 1, but not the interaction between neuropilin 1 and VEGFR2, was required for intraretinal vascular development. However, further studies are required to determine the role of neuropilin 1 in physiologic vascular development of the peripheral retina and IVNV in translational models of ROP.

### The Role of Neuropilin 2 in Models of Retinopathy of Prematurity

Neuropilin 2 knockout mice (*Nrp*2^−/−^) had significantly reduced IVNV in OIR compared with littermate wild-type mice; however, neuropilin 2 mRNA was expressed in mice raised in room air from p0 to p7 (57). Therefore, it was postulated that *Nrp*2^−/−^ mice would have reduced intraretinal vascular development compared with littermate controls. However, there was no difference in inner plexus vascular density between *Nrp*2^−/−^ mice and littermate wild-type mice raised in room air and analyzed at p7 (57). Taken together, the data suggest that neuropilin 2 is involved in IVNV but not required for intraretinal vascular development. Further studies are warranted in OIR models to determine the effect on regrowth after hyperoxia and physiologic vascular development of the peripheral retina before considering neuropilin 2 as a potential therapeutic target for ROP.

## Discussion

ROP is the leading cause of blindness and visual impairment in children worldwide. In severe cases of ROP, blindness can occur from retinal detachment caused by IVNV. Studies in OIR models that recapitulate aspects of human ROP have provided insights into VEGF signaling through VEGFRs and neuropilins in specific cell types. Experimental studies support the finding that regulating oversignaling through VEGFR2, especially in retinal endothelial cells, would not only reduce severe ROP but also facilitate normal vascular development. However, there is no suitable way to target endothelial VEGFR2 in premature infants yet. Broad inhibition of VEGF or VEGFR2 using intravitreal neutralizing antibodies or small molecules may affect signaling in other cells in the retina and affect function and structure or potentially leak into the circulation and affect developing organs. However, the use of correct dose or agent suggests that reducing the bioactivity of VEGF may have value to permit some VEGF signaling important in physiologic vascular development of the peripheral retina (10, 69). An appropriate dose of anti-VEGF may regulate overactive VEGFR2 in retinal endothelial cells, which occurs with increased ligand concentration (23, 31), without abolishing VEGFR2 signaling in endothelial or other cells of the retina.

Besides anti-VEGF, alternative approaches are being explored to prevent VEGF-mediated ROP occurrence and progression. Oxidative stresses (i.e., reactive oxygen species) have been implicated in VEGF-mediated IVNV in rodent models of ROP (70). Administration of antioxidants Cu/Zn superoxide dismutase (71) or vitamin E (72) in extremely low gestational age infants reduced the risk of ROP. However, side effects related to vitamin E (73) preclude widespread use. Also, antioxidants may fail to access the intracellular signaling mechanisms leading to pathology or counteract beneficial mechanisms of oxidative signaling. Therapeutic approaches have been considered to regulate hypoxia inducible factors, either stabilization with prolyl hydroxylase inhibitors in phase I (74, 75) or potential inhibition in phase II. It remains to be seen if the phases described in the two-phase hypothesis of ROP can be distinguished sufficiently in an individual human infant. Another treatment approach is carefully monitoring oxygen tension at birth to prevent hyperoxia-induced damage to blood vessels and reduce oxygen fluctuations that slow vascular growth to the peripheral retina (76). Additional experimental studies to regulate semaphorin/neuropilin signaling (77) might lead to future approaches in ROP. Overall, these approaches provide insights into possible therapeutic approaches to regulate VEGF-induced VEGFR2 signaling in ROP.

## Author Contributions

AR and MEH performed the literature searches and drafted and critically revised the manuscript. MEH provided funding support. All authors contributed to the article and approved the submitted version.

## Funding

This work was supported by the National Institutes of Health/National Eye Institute R01EY015130 and R01EY017011 to MEH, the National Institutes of Health/National Eye Institute F30EY032311, the National Institutes of Health/National Eye Institute P30EY014800, and an Unrestricted Grant from Research to Prevent Blindness, New York, NY, to the Department of Ophthalmology and Visual Sciences, University of Utah.

## Conflict of Interest

The authors declare that the research was conducted in the absence of any commercial or financial relationships that could be construed as a potential conflict of interest.

## Publisher's Note

All claims expressed in this article are solely those of the authors and do not necessarily represent those of their affiliated organizations, or those of the publisher, the editors and the reviewers. Any product that may be evaluated in this article, or claim that may be made by its manufacturer, is not guaranteed or endorsed by the publisher.
